# Evolving Concepts of the Schizophrenia Spectrum: A Research Domain Criteria Perspective

**DOI:** 10.3389/fpsyt.2021.641319

**Published:** 2021-02-25

**Authors:** Bruce N. Cuthbert, Sarah E. Morris

**Affiliations:** National Institute of Mental Health, Bethesda, MD, United States

**Keywords:** psychiatric diagnosis, psychiatric nosology, research domain criteria, psychopathology, schizophrenia spectrum, psychosis spectrum

## Abstract

Several trends intersecting over the past two decades have generated increasing debate as to how the concepts of schizophrenia, the schizophrenia spectrum, and the psychotic disorders spectrum should be regarded. These trends are reflected in various areas of research such as genomics, neuroimaging, and data-driven computational studies of multiple response systems. Growing evidence suggests that schizophrenia represents a broad and heterogenous syndrome, rather than a specific disease entity, that is part of a multi-faceted psychosis spectrum. Progress in explicating these various developments has been hampered by the dependence upon sets of symptoms and signs for determining a diagnosis, and by the reliance on traditional diagnostic categories in reviewing clinical research grants. To address these concerns, the U.S. National Institute of Mental Health initiated the Research Domain Criteria (RDoC) project, a translational research program that calls for studies designed in terms of empirically-based functions (such as cognitive control or reward learning) rather than diagnostic groups. RDoC is a research framework rather than an alternative diagnostic system, intended to provide data that can inform future nosological manuals. This commentary includes a brief summary of RDoC as it pertains to schizophrenia and psychotic spectra, examples of recent data that highlight the utility of the approach, and conclusions regarding the implications for evolving conceptualizations of serious mental illness.

## Introduction

The concept of schizophrenia (SZ) has elicited continual debate since the first descriptions of psychosis appeared in the middle of the nineteenth century. The nature of the concept has fluctuated across the years according to the views of the scientific zeitgeist and various schools of psychopathology, but has always persevered in one form or another (1). Within the last decade, however, advances in multiple areas of science—genomics, neuroimaging, cognitive science, and epidemiology—have begun to challenge classic conceptions of schizophrenia (2, 3).

Progress in expanding these various developments has been hampered by two major obstacles. First, disorders continue to be defined almost exclusively by sets of symptoms and signs; however, the relationships between diagnostic categories and biological or behavioral measures have proven to be modest and inconsistent, frustrating both a more comprehensive understanding of disorders and the development of more effective treatments (4). Second, research on mental disorders has been constrained by the persistence in grant review committees of a de facto requirement that hypotheses will embody DSM/ICD categories as their scientific focus, thus foiling applications proposing alternative approaches.

To address these problems, the US National Institute of Health (NIMH) initiated the Research Domain Criteria (RDoC) project in 2009 “to develop, for research purposes, new ways of [studying] mental disorders based on dimensions of observable behavior and neurobiological measures” (5). RDoC was conceived as an experimental framework to support research in psychopathology organized around basic functional domains such as cognition, motivation, and motor activity, most of which are pertinent to multiple disorders as currently defined (and may partially account for the extensive co-morbidity in current disorders).

The various elements of the RDoC framework have been described in detail elsewhere (5–7) and are briefly summarized here. RDoC is intended as an explicitly translational program: The focus is on fundamental operations of adaptive behavioral/cognitive and brain functioning (e.g., working memory, fear behavior), and psychopathology is viewed in terms of dysregulation in these systems rather than starting with clinical syndromes and trying to determine their source. A core desideratum of RDoC is to study entire dimensions of functioning from the normal range to increasingly abnormal extents, and no specific cutpoints for disorders are specified in order to encourage studies of transitions from normality to degrees of pathology. To foster such analyses, RDoC calls for study designs that include a broader range of “healthy controls,” patients with mild/subsyndromal psychopathology, and unaffected relatives of probands.

The basic dimensions of RDoC are organized in six superordinate domains of functioning (negative valence, positive valence, cognition, social processes, arousal/regulatory systems, and sensorimotor systems). Each domain contains multiple constructs, which—central to the entire framework—are defined jointly by data for a behavioral or cognitive/affective function, evidence for a neural circuit or system that plays a primary role in implementing the function, and relevance to psychopathology (8).

The domains and constructs were defined in a series of workshops attended by experts in both basic and clinical research. This process was essential for two reasons. First, it is important to communicate to the field well-validated constructs from the basic behavioral neuroscience literature that have demonstrated promise for understanding psychopathology. Second (and less evident), it is critical to provide clear guidelines for grant review. Just as established criteria for defining patient groups contributed significantly to the DSM's hegemony in study sections, examples of domains and constructs are essential to serve as standards for both applicants and reviewers in submitting and evaluating RDoC applications. Since RDoC is an experimental framework, applicants are not required to use one of the current constructs, and no claim is made that the current list of constructs is complete; in fact, a major goal of the program is to encourage research that establishes new constructs or domains, based on the premise that promoting diversity of ideas in research is the best way forward (Note that NIMH accepts DSM-oriented grant applications as always, although applicants are encouraged to address pertinent heterogeneity).

In keeping with the basic-to-clinical translational approach, RDoC focuses on relatively specific aspects of disordered functioning rather than syndromal categories. Study designs might include patients from one or more DSM/ICD categories, analyzing dimensions or subgroups in the full sample or examining selected subjects with particular characteristics (e.g., cognitive control or reward-related deficits). Participants in transdiagnostic studies are typically drawn from related areas of psychopathology, such as mood/anxiety disorders or psychotic disorders (plus comparison participants appropriate for exploring dimensions of functioning). An important emphasis concerns individual differences in psychopathology, given the heterogeneity that is now recognized for all syndromal disorders. Studies that include multiple domains/constructs are encouraged, such as the relationship of threat to attention or reward-related activity to social processes. RDoC-related research further advocates the use of multiple classes of measurement, ranging from genomics and circuit measures to behavioral and self-report, in order to seek an integrative understanding of brain-behavior relationships as they relate to particular functions.

## RDoC and the Psychotic Spectrum

The RDoC program has consistently emphasized its agnostic position with respect to disorders as defined in the DSM/ICD system: The goal is to stimulate research that can inform revisions to future diagnostic manuals, however similar or divergent to current disorders and their definitions. Recent developments in the field demonstrate novel conceptions across the entire range of psychopathology, employing various types of dimensions, clusters, and hierarchical approaches that align with the RDoC approach (9).

Research focused on psychotic disorders amply reflects this trend (10). As one expert recently explained in a publication for psychiatric professionals, “Over the last decade or so, our field has experienced a radical shift in our understanding of schizophrenia and other serious psychotic disorders, such as schizoaffective disorder and bipolar disorder with psychosis. …. Accumulating evidence indicates that psychotic disorders constitute syndromes rather than diseases *per se*. … Patients with different clinical diagnostic phenotypes … can show similar underlying patterns of cognitive dysfunction and neurobiological abnormalities” (11). Space allows only a small number of papers to be cited here as examples of RDoC approaches in the psychotic spectrum [which are treated more comprehensively in a recent chapter; (7)].

### Transdiagnostic Findings

The current interest in a schizophrenia or psychotic disorders spectrum is consistent with the kinds of trans-diagnostic mechanisms that RDoC prioritizes. There are multiple types of relevant research designs. These include overlaps between traditional diagnostic classes, such as SZ and bipolar Type 1 disorder (BPD), that are frequently used when it is difficult to examine disorder subtypes or dimensions due to the nature of measurement (as in GWAS studies). A second type of design involves transdiagnostic dimensions or gradients; these differ from the prior design in that the analyses focus on how functional domains are arrayed along one or more dimensions across two or more disorders. Finally, cluster or similar analyses use data-driven techniques to reveal groupings that cut across traditional disorder categories.

Psychiatric genetics has provided increasing support for systematically related trans-diagnostic mechanisms as sample sizes grow. Comparisons of GWAS data across disorders have shown results that are consistent with a recently-posited gradient of neurodevelopmental syndromes ordered by the extent of neurodevelopmental impairment (from most to least: intellectual disability, ASD, ADHD, SZ, schizoaffective disorder (SZ-A), BPD, major depressive disorder [MDD]; (2, 12)). Larger coheritabilities were observed for disorder pairs that were closer on the spectrum; e.g., SZ-BPD and BPD-MDD were larger than SZ-ASD or BPD-ADHD (13).

More elaborated data emerged from a study comparing eight disorders in a larger sample, resulting in three clusters of disorders based on shared loci—mood and psychotic disorders (SZ, BPD, and MDD), early-onset neurodevelopmental disorders, and compulsive behaviors (14). As the authors concluded, “… these results indicate a substantial pairwise genetic correlation between multiple disorders along with a higher-level genetic structure that point to broader domains underlying genetic risk to psychopathology. These findings are at odds with the classical, categorical classification of mental disorder.” (14, p. 1475).

A second aspect of trans-diagnostic comparisons involves dimensions that cut across disorders. For example, a recent study from the CNTRACS group employed multiple measures of performance that tapped distinct aspects of cognition (cognitive control, episodic memory, and visual perception) in a large sample consisting of individuals diagnosed with SZ, BPD, or SZ-A (15). A latent profile analysis returned a solution with three trans-diagnostic clusters of high ability (mostly indistinct from control subjects), medium performance, and low performance. The proportions of patients from the three diagnostic groups were distributed across the three ability clusters, indicating that the latter were not simply proxies for diagnosis. Confirmatory factor analysis was consistent with the presence of an underlying one-dimensional structure across the three cognitive profiles, suggesting a shared mechanism not related to diagnostic classes *per se*.

Moving toward multi-measure studies that are compatible with the RDoC approach, computational analyses that identify transdiagnostic clusters of patients illustrate the potential of empirically-derived phenotypes that align with particular biological and behavioral functions. In the exemplary B-SNIP study (Bipolar & Schizophrenia Network on Intermediate Phenotypes), investigators recruited a large sample of patients (SZ, SZ-A, or BPD with psychosis) and acquired a wide range of biological, behavioral, and clinical measures (16). A cluster analysis of factor scores from cognitive and electrophysiological measures grouped patients into three “biotypes” that cut across DSM disorder categories (as in the previous example). The first two biotypes were characterized by impaired cognitive functioning (slightly more severe in Biotype 1) but divergent sensorimotor reactivity (event-related potential responses related to simple stimuli) that was markedly blunted in Biotype 1 and hyper-responsive in Biotype 2; both measures for the third biotype were only slightly different from healthy controls. The biotypes were validated by several different measures not used in the cluster analysis, including gray matter loss as assessed by voxel-based morphometry. This study demonstrated that deriving transdiagnostic clusters based on a combination of behavioral and psychophysiological functions (cognition and perception), consistent with an RDoC approach, have promise in determining data-driven clinical phenotypes with more validity than traditional disorder classes.

### Dimensionality

RDoC emphasizes the gamut of normal-to-abnormal functioning. This aspect can be considered both in terms of cross-sectional and longitudinal studies. The latter, in this context, include trajectories of neurodevelopment from conception to risk states and overt psychopathology.

Cross-sectional discussions of psychosis dimensionality date back nearly as far as the concept of schizophrenia itself, with unresolved discussions as to whether the clinical phenomena represent one or more clinical categories, one or more dimensions, or some combination (17, 18). More recently, extensive analyses have been adduced to support replacing the schizophrenia concept with a broader “psychosis spectrum” (19) that reflects a continuous dimension of psychosis proneness from normal to abnormal (20), although also allowing for a continuous psychometric spectrum that contains one or more latent categorical structures (21).

This type of normal-to-abnormal dimensional viewpoint comports with the RDoC framework. At the same time, another RDoC principle is to remain agnostic (as with the DSM) and eschew a priori conclusions regarding the number and composition of dimensions and their clinical significance. One of the hurdles that RDoC was created to address concerns the often-modest relationships among the presence/severity of clinical symptoms and various other measures, such as cognitive tests or brain circuit activity. As noted in a recent paper on RDoC and psychosis, “… one must empirically test whether dimensionality of a symptom indicates dimensionality of a mechanism” [(7), p. 32]. In short, the field is just starting to make progress in unpacking the relationships within and across multiple neurocognitive functions, multiple kinds of symptoms, and multiple neurobiological and genetic measures—compounded by the complexities of intermixed clusters and dimensions (22). In spite of the daunting challenges, the evidence is already strong that the field is moving in positive directions.

### Neurodevelopmental Studies

RDoC places a high priority on neurodevelopmental trajectories. While the clinical high-risk state (CHR) state for psychosis is perhaps the most thoroughly researched example of a trajectory leading toward disorder (23), more recent studies have expanded the scope of neurodevelopment and functions consistent with RDoC principles. For instance, a recent study followed an unselected sample of children from age 8 to late adolescence, collecting a large number of measures including neurocognitive tests and symptoms; children who developed psychotic symptoms later in adolescence were on average 1–2 years behind typically-developing children in cognitive growth, suggesting that early cognitive impairment could be a marker for psychosis risk and that growth charting may be an opportunity for early detection and prevention (24). Another group has independently begun to implement this concept with a developmental battery of “gamified” tasks (running on a mobile e-platform) that assesses six cognitive domains in young children in India as a first step to developing normative growth curves (25).

Such promising programs are only the tip of the iceberg for neurodevelopmental studies involving RDoC (which comprise nearly half of RDoC-themed translational grants funded by NIMH). An equally important issue concerns the need to explicate neurodevelopmental changes from birth to adulthood – addressing both substantive and psychometric issues of identifying and assessing functions that emerge at various points in development, as well as relating growth trajectories to the complex effects of multiple environmental influences (26, 27).

## Summary

It is a stimulating time for research on mental disorders. The field is burgeoning with intriguing new results and new ideas – sparked by developments in genomics, neuroimaging, behavioral science, computational approaches, and many other disciplines. The RDoC initiative has been a part of this contemporary zeitgeist, enabling conversations about innovative approaches to psychopathology (28–30) and supporting research projects that represent new avenues for future directions (31–33).

These developments have accelerated progress regarding the schizophrenia (or more broadly, the psychotic) spectrum. Genomic data provide increasing support for the concept of systematic transdiagnostic components of neurodevelopmental spectra (2, 12). In this view, schizophrenia represents not so much a distinct disease as one segment of multiple broader spectra. However, the evidence is also clear that a neurodevelopmental gradient is not simply a matter of performance as assessed by the usual cognitive test batteries; it is important to consider multiple functional domains whose combinations comprise potentially significant clinical phenotypes, e.g., biotypes defined by both cognitive performance and sensorimotor reactivity (16).

A further aspect of the emerging literature, consistent with the RDoC approach, concerns various gradients from normal to abnormal functioning and how these relate to illness and dysfunction. It is now evident that some types of functional impairments are not necessarily tied to manifest clinical features. As two examples, both the B-SNIP and CNTRACS studies (summarized above) reported that patients in one of the three clusters, in spite of meeting criteria for SZ, BPD, or SZ-A, were characterized by functional performance in cognition and perception that was modestly to indistinguishably different from healthy controls (15, 16). A necessary agenda for future research is to unravel the complex relationships among the extent of such factors as genetic load, functional impairments, and clinical symptoms.

The current status of evidence about the psychotic disorders spectrum raises significant questions regarding both near-term implications for research on clinical assessment and services, and long-term directions for scientific priorities and perspectives. With respect to clinical practice, the DSM/ICD nosology continues to dominate procedures for diagnosis and treatment. However, there is increasing attention to transdiagnostic approaches for diagnosis and treatment that build upon awareness of heterogeneity and clinicians' wisdom that many (if not most) treatment plans are focused on specific problems (e.g., sleep, attention, interpersonal relationships) irrespective of formal diagnosis (34, 35), and at least one case report specifically cites the use of a transdiagnostic, RDoC approach (36). Further, some clinical programs have explicitly adopted a transdiagnostic process for assessment and treatment of first-episode psychosis in recognition of the change in diagnosis across time in many patients (37).

Regarding scientifically-driven changes in nosology, there appears to be a clear consensus that traditional disorder classes in this spectrum need to be revamped, and dozens of promising genetic, circuit-based, and behavioral findings provide clues to future classification systems. However, the nature and extent of potential changes to nosology remain far from clear, as different measurement classes and analytical techniques have yet to coalesce. There also remains the question of the granularity of concepts and measurement that are optimal for clinical use; these concerns apply across all areas—e.g., the number and combinations of specific gene abnormalities for molecularly based therapies; the count and locations of voxel-based structural abnormalities (38); or whether cognitive difficulties are best addressed at the level of broad test batteries, intermediate functional domains (e.g., executive function), or more specific operations (e.g., working memory).

A key question concerns the routes by which research advances can be implemented in diagnostic and treatment practice. Alterations to formal nosological criteria are not likely to be made soon, given conservative approaches to change in diagnostic manuals. Revisions based upon neuroscience and/or systematic behavioral data are yet more difficult to envision since they would involve an overhaul of the long-established reliance on symptoms and signs for diagnosis.

However, it is possible that rapid change may be recognized in other ways. Regulatory agencies, e.g., are well aware of the need for improved treatments and the potential for groupings and/or dimensions that manifest within or across traditional diagnostic categories. For instance, in 2016 the US Food and Drug Administration (FDA) promulgated an innovative new Drug Development Tool (DDT) Qualification program created to evaluate and approve (Qualify) such tools as “a biomarker used for clinical trial enrichment” [e.g., approval of the N170 event-related potential as a biomarker for social processing in ASD (39)] “… and a clinical outcome assessment used to evaluate clinical benefit…” (40). Further, the tools are developed in a “context of use” that represents “the manner and purpose of use for a DDT,” i.e., essentially the specific impairment to be addressed (40). Such developments could lead directly to innovative practices that advance treatment while suggesting new conceptions of clinical phenotypes that are validated inherently by their use in patient care.

## Conclusion

In sum, the notion of a psychotic spectrum is evolving rapidly, but schizophrenia—as broad concept or specific diagnostic category—remains a core aspect of contemporary psychopathology. Both general and specialty journals continue to publish large numbers of papers devoted directly to SZ, reflecting widespread support from multiple funding agencies across the world. In September, 2020 the National Institutes of Health announced the AMP-SCZ initiative (Accelerating Medicines Partnership-Schizophrenia), bringing together NIH, the US FDA, and multiple non-profit and private organizations to seek biomarkers for the diverse array of clinical trajectories and adverse outcomes observed in individuals identified as at elevated risk of psychosis. Accordingly, there seems to be little doubt that SZ will remain a central concept in mental disorders for some time to come (41). While future directions remain difficult to predict given the nascent state of the research, novel research frameworks seem likely to foster the continued expansion of research designs and integrative science—and, in turn, to stimulate more precise thinking about the nosology of SZ and the psychosis spectrum.

## Data Availability Statement

The original contributions presented in the study are included in the article/Supplementary Material, further inquiries can be directed to the corresponding author/s.

## Author Contributions

BC and SM contributed equally to the overall outline and scope of the manuscript. BC wrote the first draft. SM contributed extensive comments and edits that resulted in the final version. All authors contributed to the article and approved the submitted version.

## Conflict of Interest

The authors declare that the research was conducted in the absence of any commercial or financial relationships that could be construed as a potential conflict of interest.
